# Resistance of Wood Wool Cement Board to the Attack of Philippine Termites

**DOI:** 10.3390/insects3010018

**Published:** 2011-12-28

**Authors:** Carlos M. Garcia, Dwight A. Eusebio, Marciana R. San Pablo, Edgar M. Villena

**Affiliations:** Department of Science and Technology, Forest Products Research and Development Institute, Los Baños, Laguna 4031, Philippines; E-Mails: dwight_eusebio@yahoo.com (D.A.E.); marcianasanpablo@yahoo.com (M.R.S.P.); edgar_villena@yahoo.com (E.M.V.)

**Keywords:** resistance, wood wool cement board, *Microcerotermes*, *Cryptotermes*, *Nasutitermes*, *Gmelina*

## Abstract

The resistance of yemane (*Gmelina arborea *Roxb.)*-*based wood wool cement board (WWCB) against Philippine termites was evaluated under laboratory and field conditions. Tests were conducted following the FPRDI standard procedures in determining the resistance of WWCB against subterranean and drywood termites. Results of the laboratory tests showed that WWCB was resistant to both *Microcerotermes losbañosensis *Oshima and *Cryptotermes dudleyi *Banks. Under field conditions, WWCB was highly resistant to subterranean termites. There was no remarkable termite damage except for the normal nibbling or initial termite feeding on the board during the 8-year exposure period.

## 1. Introduction

Wood wool cement board (WWCB) is a panel product basically made of wood strands and ordinary Portland cement. *Gmelina arborea*, a fast growing hardwood species, is currently the main raw material in the manufacture of WWCB in the Philippines. WWCB has greatly reduced the cost of housing components since small diameter logs and branches unsuitable for lumber production, non-commercial wood species, and agricultural wastes of low commercial value can be utilized as raw materials in its production [1]. WWCBs are recommended for ceiling, interior partitions and walling components of houses and buildings. However, to be economically competitive, the product must meet the desired performance standards not only in terms of physico-mechanical properties but also in construction quality, such as resistance to termite attack.

Wooden components of houses and concrete structures are prone to invasion by subterranean and drywood termites [2]. The *Coptotermes vastator *Light, *Microcerotermes losbañosensis *Oshima, *Macrotermes gilvus* Hagen and *Nasutitermes luzonicus* Oshima are the major subterranean termite species that destroy houses and buildings in the country. They gain access to wooden components of the structures by constructing mud tubes in a more or less concealed manner along underground electrical cables, plumbing lines, and inside cracks and crevices of concrete posts, walls or foundations. Constant contact with the ground provides them with a source of adequate moisture necessary for their growth and development. Infestation is manifested by the presence of earthen tubes, infested wood and termite nests. On the other hand, drywood termite species *Cryptotermes dudleyi *Banks and *C. cyanocephalus* Light have no soil contact and they directly attack wood. Initial infestation is hard to detect, and when pellet-like droppings are observed, the wood components are already heavily damaged.

Several studies have been conducted on the resistance of cement composites to subterranean termites. Composites made from sengon wood (*Paraserianthes falcataria*) and betung bamboo (*Dendrocalamus asper*) were found resistant to subterranean termites *Coptotermes gestroi* Wasmann under laboratory conditions [3]. Both types of boards, however, were not completely immune to termite attack as shown by termite feeding particularly on their surfaces. The commercial cement-bonded rubberwood particleboard from Peninsular Malaysia was highly resistant to the attack of subterranean termite *C. curvignathus *under laboratory conditions [4]. Apparently, resistance of MDF, chipboard and OSB was attained by the application of borate-based preservatives or by acetylation with acetic anhydride [5,6,7].

Wood strand cement board panels exhibited good durability, structural strength, resistance to fire and high resistance to decay and attack by termites [8,9]. In the Philippines, locally produced WWCBs have been claimed resistant to termites, but scientific investigations to verify this claim are wanting [1,8,10]. Thus, this study on the resistance of *G. arborea*-based WWCB to termite attack under laboratory and field conditions was a step in this direction.

## 2. Experimental Section

### 2.1. Production of WWCB

The WWCs were manufactured following the technology developed by Forest Products Research and Development Institute (FPRDI) [1]. Small diameter (12–40 cm) *G. arborea *logs were cut into 40 cm long billets. The billets were debarked and converted into 0.5 mm × 5.0 mm × 300–400 mm wood excelsior. The excelsior were soaked for 48 h in water and air-dried prior to mixing with the required amount of cement, water and accelerator. The mixed materials were spread evenly on marine plywood caul plate and cold pressed to form 10 mm × 600 mm × 2400 mm WWCBs. The WWCBs were piled horizontally with stickers in between and conditioned for 3 to 4 weeks prior to trimming.

### 2.2. Laboratory Test (FPRDI Method)

**Figure 1:**
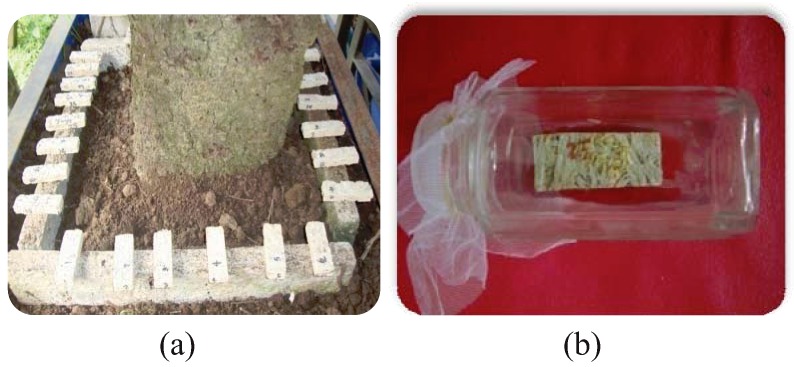
Set-up of accelerated test on the resistance of wood wool cement board (WWCB) to: (**a**) subterranean termites *M. losbañosensis* and (**b**) drywood termites *C. dudleyi*.

A nest of *M. losbañosensis *collected from the field was maintained in a termite chamber. The nest members were allowed to establish and increase their population. Fifty pieces of conditioned WWCB measuring 12 mm × 20 mm × 60 mm and *G. arborea *solid wood blocks were randomly placed over a concrete foundation and exposed to termites in the termite chamber (Figure 1a). The degree of damage on the test wood blocks and board samples was evaluated after 4 months.

Drywood termites *C. dudleyi *were collected from infested lumber in a culture chamber and carefully transferred into an aluminum tray with wood chips using a smooth camel’s brush. Theconditioned WWCB specimens and solid wood blocks of *G. arborea *were individually exposed to 100 workers and 2 soldiers inside a French-square bottle (Figure 1b). The assembled experimental bottle was incubated at room temperature and the rate of damage on the board samples was observed and recorded after 6 months.

The degree of termite damage on each board sample was evaluated based on the board’s loss in volume. The resistance of WWCB to termites was classified according to the following arbitrary ratings (Table 1).

**Table 1:** Termite-resistance ratings.

% Termite Damage	Classification (Description)
0	Highly Resistant (No evidence of termite attack)
1–25	Resistant (Slightly attacked by termites; from initial nibbling to almost ¼ of the board)
26–50	Moderately Resistant (Moderately attacked; more than ¼ to more than ½ of the board)
51–75	Slightly Resistant (Severely attacked by termites; >½ to almost ¾ of the board)
76–100	Not Resistant (Very severely attacked, more than ¾ of the board)

### 2.3. Field Test (FPRDI Method)

**Figure 2:**
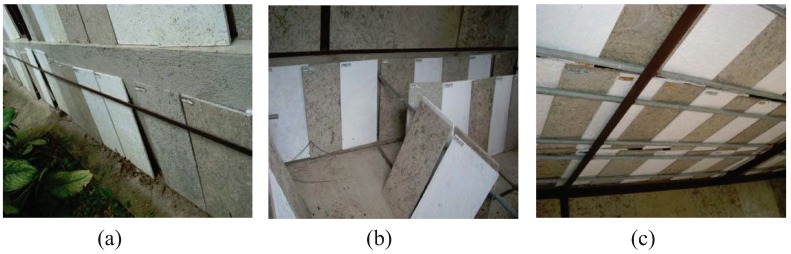
Experimental set-up of painted and unpainted WWCBs as: (**a**) siding, (**b**) paneling and (**c**) ceiling; under field conditions.

WWCBs with dimensions of 10 mm × 300 mm × 600 mm were used in the field test. Fifteen (15) each of painted and unpainted boards were installed as siding, ceiling and paneling of a pre-constructed exposure shed (Figure 2a–c). These were tested while in service, and the degree of termite damage was visually examined every 6 months for 8 years according to the above-mentioned rating scales.

## 3. Results and Discussion

### 3.1. Laboratory Test

*M. losbañosensis *extended their mud tubes from the soil in the termite chamber to the WWCB samples placed on the concrete foundation after one week of exposure. The number of tubes increased with time—a good sign of the worker termites’ foraging activities. Nibblings were observed on the board surfaces after 6 months of exposure. The mean percent damage in WWCB was 13.2% corresponding to a rating of “resistant” (Table 2). On the other hand, *G. arborea *wood blocks were moderately resistant, sustaining 37% termite damage. High termite activity was still noted in termite chamber even after the removal of board samples. The *G. arborea *wood block was moderately durable to subterranean termites, though some cases of termite attacks have been reported. The results obtained were in conformity with the *G. arborea *woodblocks exposed to subterranean termites under laboratory conditions [11]. The solid wood blocks were found moderately resistant to subterranean termites after 4 months of exposure.

It appears that termite resistance of WWCB depends on the raw materials’ inherent natural durability, the additional protection from soaking wood/wool particles in water, and the binding cement that conceals wood fibers.

**Table 2:** Mean termite damage in WWCBs and *G. arborea *wood blocks exposed to *M. losbañosensis*and *C. dudleyi*.

Test Sample	*Microcerotermes losbañosensis*		*Cryptotermes dudleyi*	
	% Damage	Resistance Rating	% Damage	Resistance Rating
WWCB	13.2	Resistant	2.1	Resistant
Wood Block	37	Moderately Resistant	4.8	Resistant

There were no comparative quantitative data on the chemical or starch content analysis of the soaked and unsoaked *G. arborea *excelsior. But soaking in water is known to reduce starch content of some materials. For instance, soaking bamboo strips in water for one week decreased their starch content from 5% to 3% and prevented beetle attack [12]. Furthermore, the cement coating of the wood excelsior seemed to provide WWCBs with supplemental protection against termite attack.

Although the WCCBs were rated as resistant composites, slight damage was seen in the interior portion, and termites entered through the gaps between wood and wool particles after the final trimming of the boards. On the other hand, termites readily attacked the surface of *G. arborea* solid wood blocks.

Interior feeding activity was demonstrated by the discharge of pellet-like materials (fecal pellets) from the board surface in the accelerated test against *C. dudleyi.* About 61% of WWCB samples and 100% of *G. arborea* solid wood blocks manifested initial termite attack after a month of exposure. However, no new pellets were discharged after a 3-month exposure, which meant the cessation of termite feeding. The WWCB and *G. arborea* solid wood blocks sustained mean damage of 2.1% and 4.8%, respectively, after 6 months, (Table 2). These results suggest that although drywood termites could bore into the WWCBs, they were unable to subsequently penetrate the substrate due to insufficient food. It was roughly estimated that 50% of WWCB samples sustained incipient damage and the rest, only 5% termite attack. The solid wood blocks were attacked for a short period, but the attack did not persist due to *G. arborea*’s inherent durability. Based on the degree of damage, the WWCBs and *G. arborea* wood blocks were classified as resistant to drywood termites.

### 3.2. Field Test

No signs of termite incidence were noted in the first 5 years of field exposure. Subterranean termites were first observed on WWCB 5½ years after field exposure (Table 3). Both *N. luzonicus *and *M. losbañosensis *extended their mud galleries from the ground line to the base of painted and unpainted WWCBs installed as exterior and interior walls. The galleries were present in approximately 50% and 33.3% of painted and unpainted exterior walls, respectively, and in 8.3% of interior walls regardless of whether painted or unpainted. Observations beneath the galleries revealed nibbling only or no termite damage on any board.

Termite incidence in WWCBs slightly decreased from 50% to 45.4% and from 33.3% to 27.3% in painted and unpainted exterior walls, respectively. Only 9.1% of painted and unpainted interior walls showed signs of termite incidence after 6 years. After 6½ years, *N. luzonicus *remained active at the base of painted and unpainted exterior walls, whereas *M. losbañosensis *became inactive on painted and unpainted interior walls.

Incidence of *N. luzonicus *was observed on painted and unpainted exterior walls only after 7 years, and that 40% of each board type did not show any damage. Termites completely disappeared thereafter. The painted and unpainted ceiling WWCBs had no termite damage for the duration of the field test.

The results showed that the presence of mud termite galleries did not always indicate termite attacks on WWCBs. The present study, therefore, demonstrated that WWCBs were not suitable food substrates for termites and were resistant to Philippine subterranean termites. These findings were relatively similar to the resistance of Indonesian composite boards under field conditions but the species of subterranean termites were not identified [13]. Subterranean termites were not able to cause infestation to locally produced WWDB due to *G. arborea*’s inherent natural durability and the cement that served as protective shield of wood excelsior.

**Table 3:** Progress in termite incidence on the WWCBs in outdoor exposure tests.

Exposure Period (Year)	% of Termite Occurrence (Types & Locations of WWCB in the Exposure Shed)					
	Painted			Unpainted		
	Exterior Wall	Interior Wall	Ceiling	Exterior Wall	Interior Wall	Ceiling
0.5	0	0	0	0	0	0
1.0	0	0	0	0	0	0
1.5	0	0	0	0	0	0
2.0	0	0	0	0	0	0
2.5	0	0	0	0	0	0
3.0	0	0	0	0	0	0
3.5	0	0	0	0	0	0
4.0	0	0	0	0	0	0
4.5	0	0	0	0	0	0
5.0	0	0	0	0	0	0
5.5	50.0	8.3	0	33.3	8.3	0
6.0	45.4	9.1	0	27.3	9.1	0
6.5	36.4	0	0	36.4	0	0
7.0	40.0	0	0	40.0	0	0
7.5	0	0	0	0	0	0
8.0	0	0	0	0	0	0

## 4. Conclusions

The study has provided technical data on the resistance of locally produced *G. arborea-*based WWCB to the attack of subterranean and drywood termites. Laboratory forced-feeding tests demonstrated that the WWCB is resistant to both subterranean termites *M. losbañosensis *and drywood termites *C. dudleyi*. Field tests also supported the observed high resistance of WWCB to *M. losbañosensis *and *N. luzonicus*. Such relatively high termite-resistance depends not only on the natural durability of raw wood materials of *G. arborea*, but also on the enhanced protection provided by the cement binder.
